# Low cholesterol is not always good: low cholesterol levels are associated with decreased serotonin and increased aggression in fish

**DOI:** 10.1242/bio.030981

**Published:** 2018-12-15

**Authors:** Ariane Aguiar, Percília Cardoso Giaquinto

**Affiliations:** Physiology Department, Biosciences Institute, Sao Paulo State University, Sao Paulo 18618-689, Brazil

**Keywords:** Plasma cholesterol, Aggressive behavior, Dominance hierarchy, Serotonergic action, Statin

## Abstract

The inverse relationship between serum cholesterol and levels of aggression led to the cholesterol-serotonin hypothesis. According to this hypothesis, low dietary cholesterol intake leads to depressed central serotonergic activity, which is associated with increased aggression. Here we present the hypothesis about the evolutionary origins of low cholesterol and aggressive behavior, investigating the relationship between low levels of plasma cholesterol and aggressive behavior in fish. We used Nile tilapia (*Oreochromis niloticus*), a species of aggressive fish with a clear dominant subordinate relation, as an experimental model. The fish were treated with statin, a cholesterol-lowering drug. Aggressive behavior, brain serotonin (5-HT) concentrations, 5-hydroxyindoleacetic acid (5-HIAA, the major 5-HT metabolite) and plasma cholesterol were analyzed after chronic administration of statin. Our results show that fish treated with statin exhibited reduced plasma cholesterol, reduced telencephalic indexes of 5-HIAA/5-HT and increased aggressive behavior compared to control fish. These results indicate that changes in plasma cholesterol may affect neurochemical processes underlying aggressive behavior in fish, suggesting an evolutionary mechanism conserved among vertebrates. Such mechanisms may be important for the control of aggression in many vertebrate species, not just mammals, as has been demonstrated so far.

## Abstract (precis)

**Summary:** Fish treated with statin exhibited reduced plasma cholesterol, reduced telencephalic indexes of 5-HIAA/5-HT and increased aggressive behavior compared with control fish. Such mechanisms may be important for the control of aggression in many vertebrate species.

## INTRODUCTION

Reduction in cholesterol has long been a desirable goal, particularly for individuals with high serum cholesterol levels and atherosclerotic disease histories. However, the optimal value of cholesterol levels is being questioned because very low levels can also affect the proper functioning of the body (Muldoon et al., 2001). Cholesterol is not the ‘bad guy’ since it is essential as a component of cell membranes, for the synthesis of vitamin D and hormones, for having an antioxidant function, as a precursor of bile salts and for working on the dynamic of neurotransmitter receptors (Shrivastava et al., 2010).

Indeed, meta-analysis suggests that deaths from coronary heart disease decrease with cholesterol-lowering treatments, but overall mortality does not, mainly due to a significant increase in deaths from external causes (accidents, suicide or homicide) (Muldoon et al., 1990; Plana et al., 2010). The increased risk of violent death is apparent when cholesterol-lowering drugs or dietary interventions have been used.

The inverse relationship between levels of serum cholesterol and aggressive behavior led Kaplan et al. (1991, 1997) to propose the cholesterol-serotonin hypothesis, which suggests that low dietary cholesterol intake leads to depressed central serotonergic activity, which in turn has been reported in numerous studies of violence. Evidence for these associations is derived from prevention trials aimed at lowering cholesterol concentrations by diet, drugs or both, from prospective epidemiological studies in which follow-ups were done in individuals with varying levels of plasma cholesterol who did not undergo specific cholesterol lowering interventions, and from clinical studies in which cholesterol and psychopathology were assessed concomitantly (for a review, see Muldoon et al., 1993). Also, reduced cholesterol esterification and disorders related to cholesterol transport have been observed in cases of depression (Linscheer and Vergroesen, 1988). Low levels of cholesterol, especially the high density lipoprotein (HDL) fraction, have been associated with several mood disorders including depression (Lehto et al., 2008).

Among cholesterol-lowering drugs, statins are one of the most prescribed drugs in the United States (Golomb et al., 2004) and worldwide. The benefits of statins in reducing the risk of coronary diseases through their action on cholesterol are substantial and well documented (Fonseca, 2005). However, there is persistent controversy related to the consequences of cholesterol reduction by statins on cognition, mood and behavior, including aggressive or violent behavior (Cummings and Psaty, 1994; Brown and Linnoila, 1990; Zureik et al., 1996; Golomb et al., 2004). Statins are lipid-lowering agents that exert their effects by inhibiting HMG-CoA reductase, a crucial enzyme in cholesterol synthesis, leading to a reduction of cholesterol.

The mechanism of action of statins occurs through the affinity of these drugs to the active site of HMG-CoA reductase (Fonseca, 2005). Statin-type drugs not only inhibit cholesterol production, but a whole range of intermediates, some of which have important biological functions. An example is the biosynthesis of coenzyme Q10 (ubiquinone, CoQ10), a cofactor required for energy production (mitochondrial ATP) and a powerful lipid-soluble antioxidant present in cell membranes (Mitchell and Redfern, 1997). Blocking the production of CoQ10, in addition to cholesterol, HMG-CoA reductase may be associated with symptoms of deficiency of this coenzyme, including malfunction of the myocardium, liver dysfunction and myopathies (including cardiomyopathy) (Goldstein and Brown, 1990).

Despite a suggested link between low cholesterol and depression, aggression and irritability, no study has investigated the possible relationship between low cholesterol and aggressive behavior in other vertebrates rather than mammals. Thus, we hypothesize about low cholesterol and aggressive behavior, using fish as a simple model, with a simpler model regarding brain-body mechanisms and behavior, in order to have clues about the complex interaction between cholesterol, serotonergic activity and aggression. Therefore, the aim of this study was to identify the relationship between the reduction of cholesterol serum, by statin treatment, and changes in the neurochemistry and aggressive behavior of Nile tilapia (*Oreochromis niloticus*). This species is a suitable model for behavior studies, being an aggressive species that form social hierarchy with clear dominant-submissive relationships and quantifiable aggression through a defined ethogram (Giaquinto and Volpato, 1997). We predict that low cholesterol will lead to depressed central serotonergic activity, decreasing serotonin (5-HT), which in turn has been reported in increased aggressive behavior.

## RESULTS

**Fig. 1.**
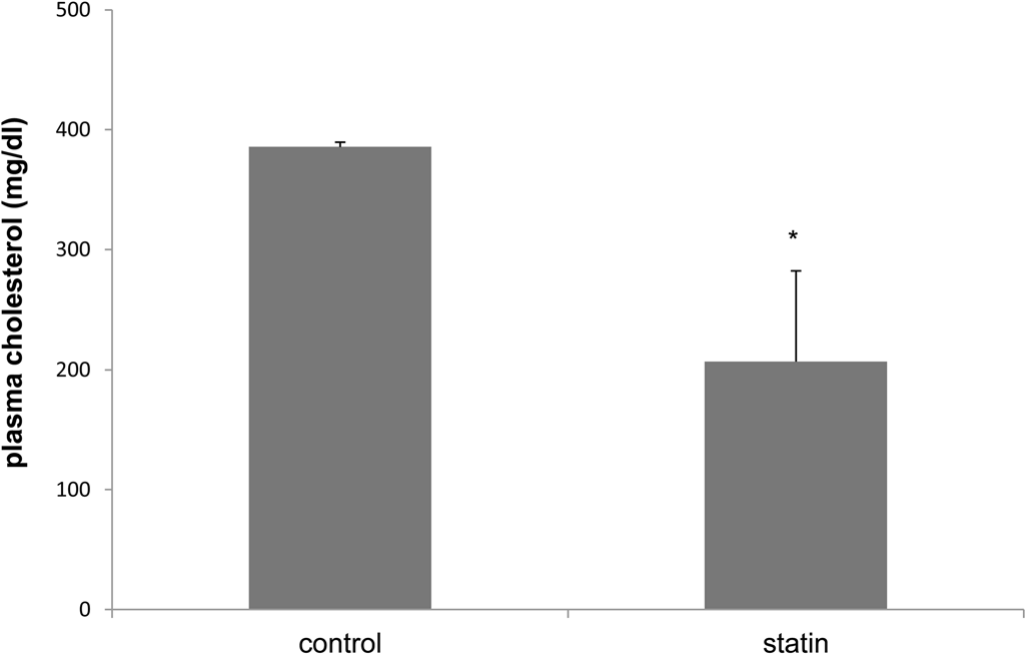
**Plasma cholesterol level (mg/dl) in Nile tilapia fish (*O. niloticus*) treated with hypolipemiant statin injection (*n*=30) and control (saline) (*n*=30) for 15 days.** Data presented as mean±s.e.m. The asterisk indicates a statistically significant difference (*P*=0.0310).

Biochemical parameters reinforced that statin was efficient to decrease cholesterol in the Nile tilapia. Statin increased liver concentrations of triglyceride (statin, 40.84±6.08 mg/g; control, 28.86±6.87 mg/g; *P*=0.0002) and decreased plasma cholesterol (statin, 206.97±25.62 mg/dl; control, 385.6±4.75 mg/dl; *P*=0.03) (Fig. 1). Statins increased enzymes alanine amino transaminase (ALT or SGPT) (statin, 8.8±10.3 U/l; control, 1.8±1.4 U/l; *P*=0.009) and aspartate aminotransferase (AST or SGOT) (statin, 61.58±22.74 U/l; control, 22.12±14.16 U/l; *P*=0.03). The analysis of hematocrit did not differ significantly (*P*=0.25) between groups.

**Fig. 2.**
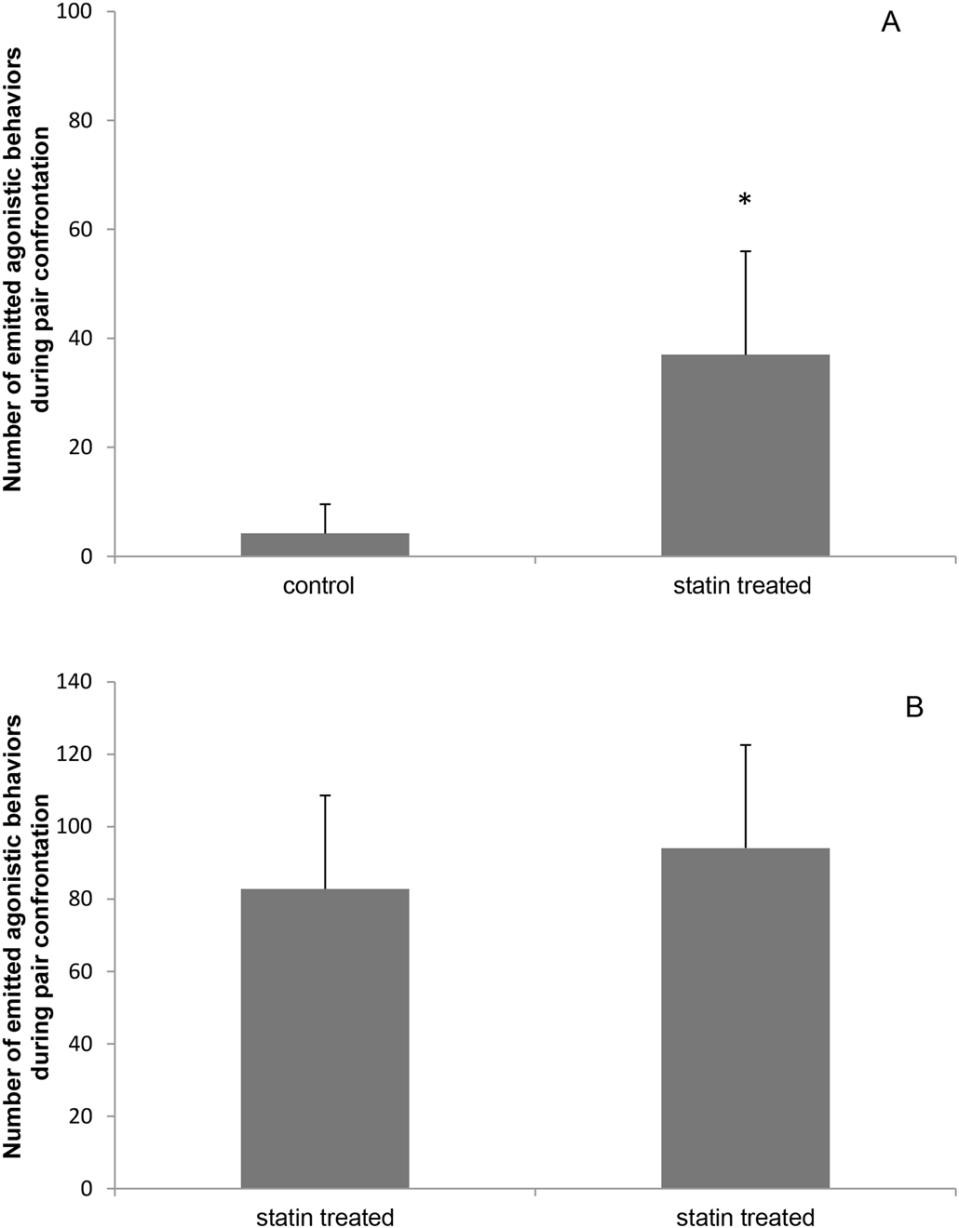
**Number of agonistic behaviors emitted by each fish in the pair (*n*=20).** (A) Control versus statin treated *O. niloticus*. Data presented as mean±s.e.m. The asterisk indicates a statistically significant difference (*P*=0.001). (B) Statin versus statin treated *O. niloticus*. Data presented as mean±s.e.m., *P*>0.05.

When pairing statin-treated with control fish, the statin-treated fish exhibited a higher number of agonistic behaviors (statin-treated=34.7±18.7; control=4.2±5.3; *P*=0.001) (Fig. 2A). When pairing was between two statin-treated fish, the number of agonistic behaviors was similar in both fish of the pair (statin-treated=94.07±28.46; statin-treated=82.76±25.82; *P*>0.05) (Fig. 2B).

Comparing the biometric parameters from the beginning until the end of the experiment, statin impaired growth (total length: statin, 0.38±0.28 cm; control, 0.58±0.27; *P*=0.03). However, statin did not affect food ingestion (statin, 3.03±1.20 g; control, 3.36±1.02 g; *P*=0.23) and body weight (statin, 3.06±0.97 g; control, 3.89±1.34 g; *P*=0.3).

The calculation of hepatosomatic index (HSI) showed no statistically significant difference (*P*=0.4782) between the groups regarding the relationship between liver weight and body weight of fish (treatment group=24.10±6.06; control group=23.95±7.65).

**Fig. 3.**
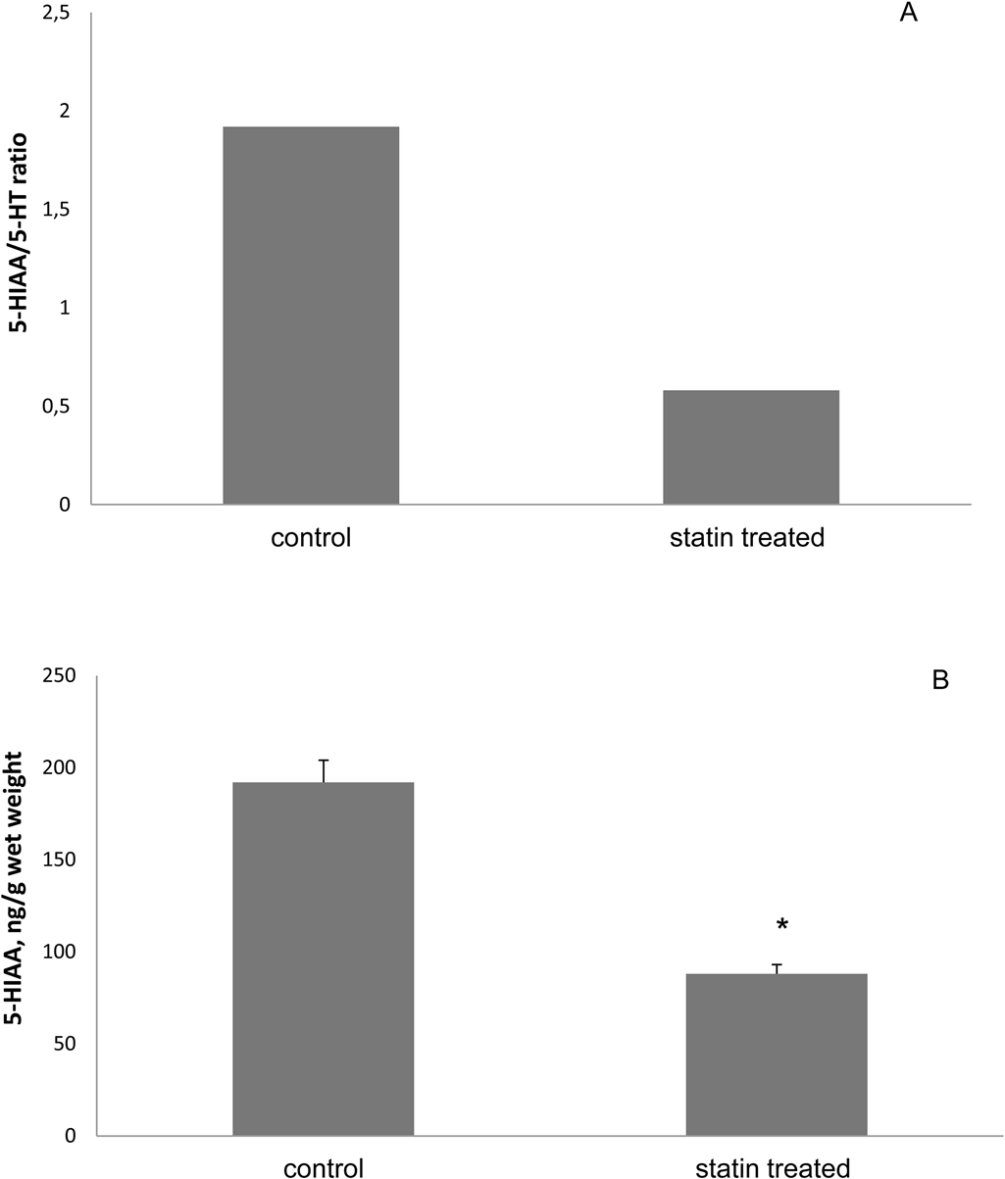
**Comparison of concentration levels and ratios between statin treated and control *O. niloticus*.** (A) Brain 5-hydroxyindoleacetic acid (5-HIAA) concentrations after statin treatment comparison to controls. Data are presented as mean±s.e.m. *P<0.001. (B) Whole brain 5-HIAA/5-HT ratio was significantly lower in treatment groups. All data are presented as mean±s.e.m. *P<0.003.

Telencephalic 5-HIAA concentrations were lower in fish treated with statin (*P*<0.001) (Fig. 3A). 5-HIAA/5-HT ratios decreased in the telencephalon of treated fish and differed significantly from controls (*P*<0.003) (Fig. 3B).

## DISCUSSION

This is the first study to show that a low cholesterol level increases aggression in fish, corroborating the cholesterol-serotonin hypothesis so far only investigated in humans and other mammals. Statin-treated fish had reduced cholesterol, which increased the number of emitted aggressive behaviors and DI. Moreover, there was a correlated reduction of central serotonergic activity. This supports that the association between low cholesterol and aggression can be a causal phenomenon.

Food and sex are the two major causes of aggression. Other causes such as territoriality, social ranking, self-defense, mate guarding and maternal aggression are all related to the two basic needs of food and reproduction. Effective aggression can result in more access to food or better mating opportunities. However, aggression has an energetic cost as well as increased risk of injury. Therefore, when there is no need for aggression, or aggression is unlikely to be effective, it has to be controlled.

Studies show that dominant and aggressive individuals have higher levels of testosterone/estradiol, increased basal levels of corticosterone (Sapolsky, 1992; Blanchard et al., 1993) and higher plasma glucose levels (Shively and Kaplan, 1984. Here we demonstrate that low cholesterol levels can also be added to characterize the aggression biochemical profile.

### Biochemical parameters

#### Tryacilglycerol indexes (TG)

Here, the fish treated with statin showed increased levels of hepatic tryacilglycerol. This effect can be explained by the accumulation of the substrate acetyl-CoA, since the enzyme HMG-CoA reductase had its share reduced in fish treated with statin. When the acetyl-CoA accumulates, it follows another route of metabolism, being converted in Malonyl-CoA and subsequently in FFA-CoA, which when added to glycerol-P is converted to tryacilglycerol.

The literature is still relatively controversial regarding this finding. Some show that statin drugs, in their therapeutic doses, significantly decrease the concentration of TG (Branchi et al., 1999). However, there is a relationship between physiopathology and high levels of hepatic TG (Zago et al., 2010), which would also explain the high TG levels found in fish treated with atorvastatin, as they showed an impairment of liver function. This biochemical parameter can also be an indirect indication of cholesterol level and aggressive behavior.

#### ALT and AST enzymes

The results of this study show that fish treated with statin showed increased levels of ALT and AST enzymes. All together, these disrupted biochemical parameters related to low cholesterol levels can work as signals for fat-depleted organisms, the behavior output of which is aggression. Higher blood enzymes could result in increased plasma enzyme activity in two ways, either due to leakage from injury or stimulation that accumulates the enzyme and as a result the amount that is likely to leak. Besides, it is important to remember that the stimulation may occur as a result of the organism's mobilizing energy sources, including amino acids, which handle stress and restore damage (Masola et al., 2008). In our study, stress could be trigged by aggressive behavior and its related high costs.

#### Neurobiological mechanisms

The basic mechanisms linking cholesterol and serotonergic activity have not been fully elucidated. One argument is that lowering cholesterol can affect the composition and structure of the cell membrane, which in turn may induce secondary changes in the receptor bound to the membrane and thus influence neurotransmitters and their emotional and behavioral effects (Muldoon et al., 1993). Also, cholesterol participates in the fluidity of neuronal membranes, synaptic vesicles and formation (Mauch et al., 2001; Pfrieger, 2003; Koudinov and Koudinova, 2002), as it can influence and has the ability to bind receptors (Burger et al., 2000).

Fat intake has shown to influence fatty acid uptake in the brain (Wang et al., 1994), the composition of cell membranes (Clandinin et al., 1983) and the neuronal activity of mitochondrial MAO (Crane and Greenwood, 1987). Also, Mullen and Martin (1992) showed that there were higher levels of serotonin in the area Raph6 after consuming a diet rich in saturated fats compared with a diet high in polyunsaturated fat (in just 4 days). Together with our results, these observations indicate that changes in dietary fat and/or inhibiting formation of cholesterol can affect both the structure of neural cells as well as neurochemical processes and thus could provide a mechanism for the effects on behavior and mood.

The findings of this study may indicate that changes in plasma cholesterol (via a lipid-lowering drug) can affect the structure of cells and neurochemical processes and thus could provide a mechanism for the effects of aggressive behavior. One argument is that lowering cholesterol can affect the composition and structure of the cell membrane, which in turn may induce secondary changes in the receptor bound to the membrane and thus influence the neurotransmitters and their emotional and behavioral effects (Muldoon et al., 1993), possibly through the mediation of the central serotonergic system.

The diversity of aggression in terms of motivation, behavior and function are accompanied by extensive neural pathways and neuroendocrine. One of the most studied neurotransmitters is 5-HT. In recent decades, it has been shown that there is a relationship between low concentrations of 5-HT in the brain and expression of aggressive behavior (Wallner and Machatschke, 2009). Thus, we may suppose that the increased aggressiveness of fish treated with statin is related to decreased serum cholesterol and its cascading effect on neuroendocrine and behavioral pathways.

### Corroborating cholesterol-serotonin hypothesis in nonhuman vertebrates

Kaplan et al. (1997) theorizes that lowering serum cholesterol results in the reduction of central serotonergic activity, which in turn increases aggressive behavior. This hypothesis is biologically and evolutively plausible; natural selection may have altered the behavioral effects of reducing serum cholesterol (Erickson, 1997).

The fluctuation between periods of famine and plenty, which characterizes the diet of early humans, may therefore have resulted in the evolution of a mechanism related to cholesterol lowering diet and an increase in aggressive behavior (risk, impulsivity, hunting), which in turn resulted in increased intake of cholesterol (Kaplan et al., 1997). Another study links low cholesterol levels and low concentrations of serotonin in men, in a comparative study between men and women, predispose them to violent behavior and risk (Markianos et al., 2010).

In conclusion, this study indicates that changes in plasma cholesterol levels may trigger serotonin depletion and related behaviors, such as increased aggression. Also, this study suggests that this is a conserved trait among vertebrates.

## MATERIALS AND METHODS

### Experimental design and procedures

In order to test the role of low cholesterol in aggressive behavior, fish were chronically treated with statin, a cholesterol-lowering drug. We then analyzed aggressive behavior, brain serotonin concentrations, 5-HIAA, the major 5-HT metabolite, and plasma cholesterol. Sixty juveniles of Nile tilapia (*O. niloticus*) with initial weight of 30.13±6.62 g and standard length of 9.93±0.76 cm were selected from a stock that originated from a fish farm and were acclimated to the laboratory conditions for 30 days in 500 l tanks before the experimental procedures. The fish were housed in individual 18 l aquaria and were allowed a week for acclimation prior to the experiment, in order to reduce the effects of pre-established hierarchy and handling stress and to ensure that all fish resumed feeding (indicator of well-being). The aquaria were supplied with a constant aeration system. A controlled photoperiod of 12 h light:12 h dark was applied. Following acclimation, the fish were fed daily with commercial pellets at 09:00 with a ration equivalent to 3% of fish mass. Food was available to fish for a period of 30 min, after which the remains were removed and quantified to determine the amount of food ingested. Twice a week the aquaria were siphoned to remove feces and approximately 20% of the total volume of water was replaced. Water quality parameters were monitored regularly: values ranged for pH (7.1–8.0), dissolved oxygen (5.6–6.7 mg l^−1^), ammonia nitrogen (0.12–0.20 mg l^−1^), nitrite nitrogen (0.33–0.58 mg l^−1^) and for alkalinity as CaCO3 (235–250 mg l^−1^).

Maintenance and experimental procedures were approved by the Ethics Committee of the State University of Sao Paulo [Instituto de Biociências – Comissão de Ética na Experimentação Animal (CEEA), protocol number: 151].

#### Drugs and administration

Statin was used as a hypolipemiant (Lipitor^®^; Pfyser, Guarulhos, Brazil) because it is widely prescribed. Statin drugs are inhibitors of 3-hydroxy-3-methylglutaryl coenzyme A and cause partial and reversible inhibition of the enzyme HMG CoA reductase, a key enzyme in cholesterol endogenous synthesis route. Stock solutions of statin were prepared by dissolving the components in methanol concentrations of 400 µg/ml. All solutions were stored at 4°C and were stabilized for at least 4 weeks (Bahrami et al., 2005). Since there are no studies relating statins and fish behavior and plasma parameters, the dose was set to according with the prescription to doses in human. Therefore, based on the stipulated amount of 80 mg for a 70 kg adult, the dose was adjusted as approximately 0.1 mg for a 50 g fish.

Statin was administered during the first 15 days of the experiment (*n*=30). The route of administration was intramuscular, and therefore required a hydrosaline sterile dilution, used also as control (*n*=30). For application, fish were anesthetized with benzocaine (Fagron Iberica, Terrassa, Spain) diluted in water (12 g/l), and after a rapid procedure (30-45 s), returned to aquaria. The injection was applied alternately between the left and right sides of the fish. Fish were marked individually (small cut at the fins) to distinguish between treated and control fish. At the end of each confrontation test, animals were returned to their home aquarium and continued to receive food daily, and statin/saline was applied every second day.

Fish biometry was taken at the beginning and end of experiments in order to check statin effect in growth and weight.

### Behavioral test

For the behavioral test, contests were staged between pairs of fish of the following type: statin-injected versus saline-injected (*n*=10 pairs), statin-injected versus statin-injected (*n*=10 pairs) and saline-injected versus saline-injected (*n*=10 pairs). The contests were conducted in a neutral aquarium, and lasted 25 min (long enough to cover the peak of statin action). The contests were video-recorded and the occurrence of agonistic behavioral acts from each individual fish of the pair was noted by two observers that were unaware of fish fin marks (double blind design). Confrontations were continuously counted during the period of observation and the frequencies and durations of aggressive acts were recorded.

### Social dominance hierarchy

Dominance relationships were defined by the Dominance Index (DI), previously used by Gómez-LaPlaza and Morgan (1993), Oliveira and Almada (1996) and Bailey et al. (2000). The DI of each fish was calculated as the ratio of emitted attacks/(received+emitted attacks), ranging from 0 to 1; it was expected that the DI of dominant fish would be higher than that of the submissive opponent. To evaluate the stability of hierarchy, we calculated the differences between the DIs of the paired fish (DI dominant−DI subordinate). Values close to 1 showed a high stability and low values, close to 0 showed a low stability. The dominance hierarchy was also qualitatively identified after matching the pairs, considering: (a) body color (dominant fish are clear and bright and subordinates are darker, with dark vertical stripes); (b) who emits attacks (dominant fish emit and subordinates evade when hierarchy stabilizes); (c) territory occupancy and (d) position of the dorsal fin (most often upright on the dominant fish).

After 15 days, a second behavioral test was conducted with the same pairs using the same methodology described above. At the end of each confrontation, fish weights and lengths were measured for comparison with the initial measurements.

### Blood and liver analyses

At the end of behavioral tests, fish were anesthetized with benzocaine (1 g/15 l water) and a blood sample was removed by cardiac puncture with a 1.0 ml syringe. Blood samples were used to determine hematocrit and plasma cholesterol. The blood samples were placed in eppendorfs and centrifuged at 3000 rpm for 5 min to remove the plasma, and subsequently stored in a freezer at −80°C. A percentage of hematocrit was determined using a micro hematocrit centrifuge.

The collected blood was also used to determine plasma concentrations of the enzymes alanine amino transaminase (ALT or SGPT) and aspartate amino transaminase (AST or SGOT). Usually, the use of statin increases these enzymes and, in fish liver enzymes, are considered important as biomarkers, since they provide the general framework of the physiology of the animal and its health. ALT and AST are examples of where the variation of aminotransferase activity in plasma may be a sensitive indicator of cell damage in the membrane of certain organs, especially the liver. The plasma activity of these substances was determined with the UV kinetic method, using commercial kits (Transaminase AST/SGOT, Transaminase ALT/SGPT Katal^®^, Sao Francisco, Brazil) on biochemical equipment (Cobas Mira Plus; Roche Diagnostics, Basel, Switzerland) at 37°C.

In order to verify the effectiveness of the drug used, we measured plasma cholesterol levels. For this, we used the Enzyme Laborclin^®^ method, which uses reagent (Cholesterol Bioliquid^®^, Laborclin, Pinhais, Brazil) and a Cholesterol Standard solution. The samples were homogenized and placed with the reagents according to the manufacturers. The tubes were identified as White, Standard and Testing, and subsequently placed in a water bath (37°C for 5 min). The reading was spectrophotometer (Shimadzu, Kyoto, Japan) The reading was performed on a spectrophotometer (Shimadzu, Kyoto, Japan) with wavelength set to 500 nm (green filter 500-520 nm). To determine the concentration of total cholesterol in the sample, the following formula was used:

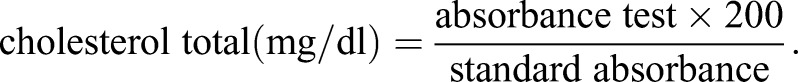


After blood collection, fish were killed by a blow to the head, their weight and standard length were measured and their livers were removed, weighed and sectioned into two parts, one for histological analysis and another to determine triglyceride levels.

For histological analysis, liver tissue was dehydrated and cleared by placing in an alcohol bath (70%–100%) for 1 h; with three changes of xylene for 1 h, then was embedded in Paraplast (Sigma-Aldrich) and cut with a 7 mm thick microtome. Coloration was carried out using changes of xylene (70% ethanol) and 5 min for each bath with a Hematoxylin and Eosin stain, or Permount and then cover slip using Paramount or Canada balsam (Thermo Fisher Scientific).

In order to determine the size differences between animals, we calculated the HSI, which is the ratio of liver mass (mg) to total body mass (g). For determination of triacylglycerols (TG), hepatic tissue samples were homogenized in a mixture of chloroform/methanol and the concentration of TG was determined from enzymatic hydrolysis (Lopes-Virella et al., 1977).

### Analysis of brain 5-HT neurochemistry

#### Tissue sampling

At the end of each experimental period, fish were decapitated, the brain of each fish was rapidly removed and the telencephalon was separated. After fast decapitation, the brain samples were frozen in liquid nitrogen and kept at −80°C. Telencephalon lobes were weighed and homogenized using an ultrasonic disintegrator (Hielscher, Teltow, Germany) in a homogenizing reagent [4% perchloric acid (PCA) containing 0.2% EDTA and 40 ng ml^−1^ dihydroxybenzylamine hydroxide (DHBA) solution] (Sigma-Aldrich). The samples were then centrifuged at 10,000 ***g*** for 10 min at 4°C. The supernatants were analyzed by high-performance liquid chromatography (HPLC) with electrochemical detection to quantify the concentration of 5-HT and its metabolite 5-HIAA. 5-HT and 5-HIAA content were quantified by comparing them with standard solutions of known concentrations and corrected for recovery of the internal standard using HPLC software (ChromNAV 2.0 HPLC, Easton, USA).

### Statistical analysis

The data were checked for normality and homoscedasticity using the Shapiro–Wilk test and Hartley’s (Fmax) test respectively, and then compared by *t*-test to identify behavioral differences between control and animals subjected to statin. Significant differences between the DI of the opponents were considered as those above 0.6.

To compare the blood biochemical and neurochemical parameters of treated fish and controls, we submitted the results to a *t*-test.
